# Comparative Analysis of the Performance of Complex Texture Clustering Driven by Computational Intelligence Methods Using Multiple Clustering Models

**DOI:** 10.1155/2022/8449491

**Published:** 2022-09-29

**Authors:** Jincheng Zhou, Dan Wang, Lei Ling, Mingjiang Li, Khin-Wee Lai

**Affiliations:** ^1^School of Computer and Information, Qiannan Normal University for Nationalities, Duyun 558000, China; ^2^Key Laboratory of Complex Systems and Intelligent Optimization of Guizhou, Duyun 558000, China; ^3^Key Laboratory of Complex Systems and Intelligent Optimization of Qiannan, Duyun 558000, China; ^4^School of Mathematics and Statistics, Qiannan Normal University for Nationalities, Duyun 558000, China; ^5^Department of Biomedical Engineering, Faculty of Engineering, University of Malaya, 50603 Kuala Lumpur, Malaysia

## Abstract

Traditional texture cluster algorithms are frequently used in engineering; however, despite their widespread application, these algorithms continue to suffer from drawbacks including excessive complexity and limited universality. This study will focus primarily on the analysis of the performance of a number of different texture clustering algorithms. In addition, the performance of traditional texture classification algorithms will be compared in terms of image size, clustering number, running time, and accuracy. Finally, the performance boundaries of various algorithms will be determined in order to determine where future improvements to these algorithms should be concentrated. In the experiment, some traditional clustering algorithms are used as comparative tools for performance analysis. The qualitative and quantitative data both show that there is a significant difference in performance between the different algorithms. It is only possible to achieve better performance by selecting the appropriate algorithm based on the characteristics of the texture image.

## 1. Introduction

Pattern recognition research places a significant emphasis on the study of image classification. The labeling of the image samples with the appropriate categories after they have been categorized is its responsibility. The characteristics of picture samples serve as the foundation for classification [1], with texture being one of the most important features to use when attempting to characterize image detail information [2]. Shape, color, texture, and other low-level features as well as more complicated high-level feature information are some examples of the characteristics of picture samples. As a consequence of this, the investigation of texture image feature extraction and classification method is of significant significance, both in terms of theory and practice.

Images with a texture can accurately reflect the surface characteristics of the objects or scenes they depict, and they are a visual element that is both common and important. Because of this, the extraction and recognition of texture pattern characteristics that are included in texture images has always been an important study direction [3], particularly in the fields of image understanding, pattern recognition, and computer vision. The primary goal of texture classification is to extract discriminative texture features from texture images. Once these features have been extracted, some type of distance measurement and classifier are applied to determine the category of the texture image. The most important aspect of research that goes into texture image recognition is the process of extracting the features of textured images. Even with powerful classifiers, it can be difficult to obtain decent recognition results if the derived texture features lack the ability to differentiate between different types of textures. Extensive research has been done on texture images by academics, primarily in the areas of texture feature extraction, texture image segmentation, texture image classification, recognition, and others. The process of identifying textures requires the extraction of important features from the textures [4]. The accuracy of the classification of texture images is significantly influenced by the performance of the texture characteristics. Photos of textures that have desirable textural qualities are simpler to categorize. Bad texture features, on the other hand, make it difficult to classify texture images, which not only take a long time to classify but also have a poor classification effect. This is because bad texture features make it difficult to distinguish between different types of textures. In addition to the properties of the texture, the classification methods that are used have a significant impact on the result of the classification of texture images [5]. Methods that are effective in classification cut down on the amount of time spent on the process while simultaneously raising both classification efficiency and accuracy [6]. Therefore, the extraction of features from texture images and the development of classification schemes for those images are essential components of texture image analysis.

Over the course of the past half century, academics from both the United States and other countries have engaged in a substantial amount of research on various textural aspects. As our knowledge of texture images expands, a number of different algorithms for the extraction of texture picture features are presented and have gained widespread use. The gray-level co-occurrence matrix, also known as GLOM [6] is the most representative approach to the process of extracting features from texture pictures. The extracted texture feature has a strong effect on classification when applied to photographs with regular textures. There will be some correlation and co-occurrence between the two pixels at some distance because the texture image is generated by the periodicity change of gray distribution in spatial position. This means that there will be some distance between the two pixels. The GLCM algorithm analyzes an image's texture in order to characterize its features by locating gray-level co-occurrences in space.

The Markov Random Field (MRF) [7–9] and the Fractal Dimension [10–12] are two approaches to texture segmentation that have stood the test of time. Because the MRF technique considers the texture picture to be a two-dimensional random process and assumes that each pixel's gray-value is solely related to that of the surrounding pixels, the texture image is modeled as a two-dimensional MRF model [13, 14]. This is because the MRF technique assumes that each pixel's gray value is solely related to that of the surrounding pixels. Because the parameters of the MRF model can explain both the intensity and the direction of the texture image, the features of the texture image correspond to the parameters of the MRF model. The result of this is that the process of extracting features from a texture image is the same as the process of estimating the model parameters in the MRF approach, and the estimated parameters can be used to characterize the features of the texture image. To put it another way, the MRF technique utilizes the two-dimensional random process that is commonplace in conventional signal analysis in order to describe the characteristics of image textures [15]. The primary objective of the fractal dimension approach, which is classified as a method of structural analysis, is to investigate the structure and morphology of texture textons at various scales in texture photographs. Many times, rather than using an integer, a fraction will be used to express the fractal dimension. Researchers have shown that the roughness of an image correlates with its fractal dimension. When the corresponding image is more jagged, the fractal dimension will be larger; conversely, when the matching image is smoother, the fractal dimension will be smaller. Fractal dimension can be used to represent the roughness of a texture image because texture pictures exhibit self-similarity and varied roughness at different scales. This means that fractal dimension can be used as the characteristic parameter for the classification and segmentation of texture images [16]. Another well-known approach to extracting features from texture images is known as the Gabor filtering method. It is analogous to the wavelet transform method of investigation. Both of them are capable of carrying out analyzes of images at multiple resolutions. As a consequence of this, another name for it is the Gabor wavelet approach. The Gabor filtering method takes into account the textural properties of different scales as narrow-band signals. The method then extracts the features of these narrow-band signals using filter-banks that have varying center frequencies and bandwidths. Over the past few years, the Local Binary Pattern (LBP) and its improved algorithm have become the method of choice for extracting texture picture features the majority of the time. The LBP technique is the method that is utilized the most frequently for the purpose of texture feature extraction because the extracted texture features not only have excellent texture classification accuracy, but also have simple theory, efficient implementation, and invariance to monotonous lighting changes. The fundamental concept behind the LBP algorithm is to encode the local neighborhood features and then calculate the histogram of these encoded values as the texture image's feature description. This process is repeated until all of the local neighborhood features have been encoded.

As the technology for texture image classification improves, more difficult and complex texture image processing tasks are assigned, but the feature extraction methods that have been around for a long time have noticeable bottlenecks. In the field of texture picture feature extraction, the method that is capable of self-learning has started to become more popular. The concept of deep learning refers to a model of neural networks that simulates the layered extraction of human brain functions. It employs a multi-layer network topology and learns from a huge number of training examples, which enables it to develop a very complicated model and complete an extremely challenging task. As a result, it can automatically extract discriminative characteristics relevant for classification from samples. Although deep learning has made significant advancements in texture classification, the traditional method has reached a high level of maturity after decades of development and is frequently used in engineering. This is in contrast to the situation with deep learning, which has made significant advancements in texture classification.

This study will focus primarily on the analysis of the performance of a number of different texture clustering algorithms. In addition, the performance of traditional texture classification algorithms will be compared in terms of image size, clustering number, running time, and accuracy. Finally, the performance boundaries of various algorithms will be determined in order to determine where future improvements to these algorithms should be concentrated. Before moving on to the actual process of extracting the texture features, the structure arrangement of this paper begins by performing a Gabor filtering preprocessing on the grayscale image. After that, the texture image is clustered and segmented utilizing a number of different clustering algorithms; the NMI and RI indicators are calculated by comparing the obtained label value with the ground-truth label; and finally, the results of the texture segmentation are compared and analyzed qualitatively and quantitatively.

## 2. Basic Feature Analysis of Texture Image

**Figure 1:**
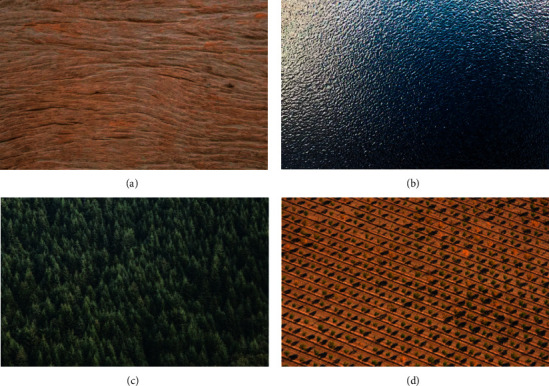
The sample of single-texture images. (a) Wood; (b) Lake; (c) Forest; and (d) Farmland.

Texture images can be found anywhere in the environment of day-to-day life, and it is not difficult to obtain or identify them, as demonstrated in Figure 1. These features of the texture pattern can describe the fundamental characteristics of an object's surface or structure, and they are a very important aspect of human visual perception that contributes to cognitive function. Natural texture, artificial texture, and a combination of the two are the most common types of textures found in real-world images of textures. Many images, such as cloth, wood, and forests, among others, have many similar repeated patch-units that are regularly distributed. These kinds of images are frequently referred to as texture images, and the repeated regular distribution of similar units is frequently referred to as the texture's features.

The textural aspects of an image sometimes take the form of patterns such as spots, grids, stripes, rings, and other similar designs. These patterns are used to characterize the spatial distribution and spatial relationship between the gray levels of an image. It is a regular and thorough reflection of a huge number of patch-units that have properties that are either comparable or identical to one another. Generally speaking, the surface characteristics of many items have very distinct differences in texture. For instance, the texture of the forest is more granular than the texture of the farmland, the texture of the farmland is less visible than the texture of the lake, and the rate of color change is more gradual. The resolution of an image has a significant impact on the characteristics of its texture. For instance, high-resolution pictures are able to portray the details of small ground objects quite well, but the features of the textures themselves are not readily apparent. Images of low resolution make the details of textures and patterns much more apparent. Texture characteristics, in comparison to geometric features and gray features, hold a greater amount of information. It has found widespread application in a variety of domains. As a result, the categorization and recognition of texture images have emerged as significant tools for the human visual system to use in its quest to understand its surroundings. The extraction of features from a texture image is both an essential step and an important part of the process of comprehending and recognizing a texture image.

**Figure 2:**
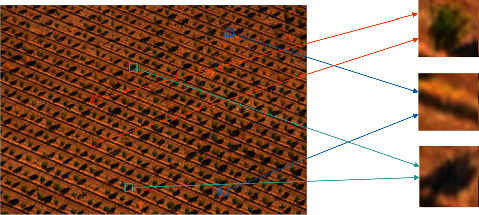
Texture feature and its textons.

It is challenging for scholars to provide an exact and consistent description of texture as well as a mathematical model of texture as illustrated in Figure 2. This is because there is a vast diversity and complexity of texture patterns or patch-units contained in texture photographs. As of recent times, there is no definition of texture that is generally recognized by everyone. Despite the absence of a standardized definition of texture and a mathematical model for describing it, there is a general agreement among people on the following qualities of texture images: textons are the fundamental unit of visual perception in texture images, and their appearance is characteristically repetitive. Textons can appear in a texture image in either a regular or random pattern, depending on the type of texture being created. Because of this, the existence and repeatability of texton is considered to be the most important quality of a texture image. When viewed from this angle, a texture image can be understood to represent the culmination of a process in which texton is arranged or distributed in accordance with a set of predetermined guidelines. The detection of texture textons and their repeatability has become the main study content of texture image feature extraction and classification because diverse texture textons and arrangement rules can combine to generate thousands of distinct texture images. In addition, texture textons typically depict regional aspects of the texture images. Therefore, in order to properly grasp texture images, we should not think of texture textons as a point process but rather as regional features that fall within a particular range. This is the approach we should take when analyzing and comprehending texture images.

**Figure 3:**
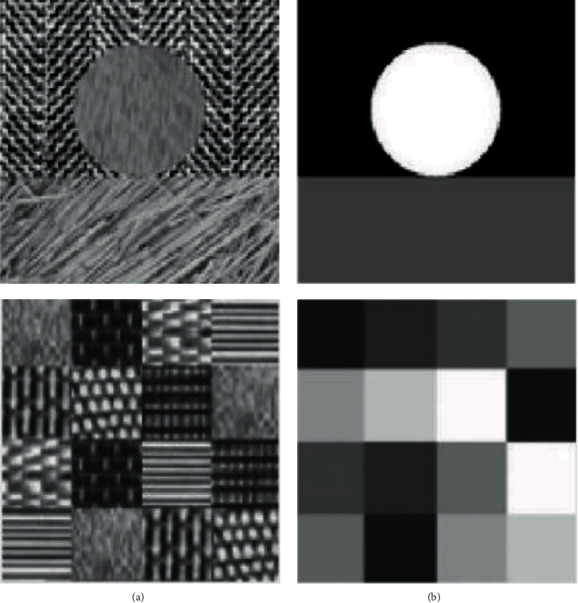
Texture images with multiple categories and its ground-truth. (a) Multi-texture images. (b) Ground-truth.

Because each of the examples that are shown in Figures 1 and 2 only contains a single texture, it is very simple to analyze the properties of each of the textures using these figures. Actual photographs, on the other hand, exhibit a diverse range of surface qualities. Mountains, rivers, farmlands, and lakes are all examples of different types of textured regions that can be found in remote sensing photographs. Nontexture regions are the parts of an image that have only minor or no shifts in grayscale, whereas texture regions are the parts of an image that have significant shifts in grayscale. Texture regions are referred to as the parts of an image that have significant shifts in grayscale. It is difficult to extract different types of texture features using methods such as threshold segmentation and others due to the fact that this is the case. In Figure 3, you can see a picture that contains multiclass textures, as well as the findings of the ground-truth classification that correspond to those results (b). If one wishes to successfully realize the texture description of a texture region, the texture clustering approach is an absolute necessity. A texture feature is a feature that has the ability to successfully differentiate between various different textures. The quality of its extraction has an effect on the accuracy of the results obtained from texture classification, texture identification, and any other subsequent texture image processing. This is because the accuracy of these outcomes is dependent on the extracted texture. A good texture feature should have the advantages of small computation, small feature size, and strong discrimination capacity, and it should also be able to be employed in engineering. These are the characteristics of a good texture feature. The traditional method of texture classification has progressed to a more developed stage and is widely applied in engineering.

After decades of development, the traditional method has reached a high level of maturity and has seen widespread application in the field of engineering. Researchers have made significant strides in the area of texture feature extraction and have proposed a number of different methods to extract texture features. These methods include fuzzy subspace clustering, K-mean, meanshift, Gaussian mixture model, LBP, fractal model, and wavelet. The research on texture feature extraction methods is booming and has great prospects, but the traditional methods have limitations as a result of the hazy definition of texture and the high complexity of texture. This is due to the fact that texture can be difficult to define. This paper will focus primarily on the analysis of the performance of various texture clustering algorithms, as well as the comparison of that performance to the performance of traditional texture classification algorithms in terms of image size, clustering number, running time, and accuracy. Additionally, the performance boundaries of various algorithms will be determined in order to locate the focus for subsequent improved algorithms. In the following section, we will discuss a number of traditional texture clustering algorithms in order to compare and contrast their performance with other algorithms in the experimental section.

## 3. Typical Texture Clustering Algorithms

### 3.1. K-Means

The K-Means algorithm is a method of unsupervised learning and clustering that is founded on the concept of partitioning. The Euclidean distance is the standard indicator that is utilized in the process of measuring the degree of similarity between different data samples. The distance between the data objects has an adverse effect on the similarity, which is expressed as a negative proportion. The greater the degree of resemblance, the closer together the points fall. The procedure requires the starting number of clusters, *k*, as well as the initial cluster centers to be specified in advance. The position of the cluster center is continuously updated, and the sum of squared error (SSE) of the clusters is continuously lowered, all in accordance with the degree to which the data item and the cluster center are comparable to one another. The clustering process is complete and the ultimate outcome is attained when either the SSE ceases to experience any changes or the objective function converges.

**Figure 4:**
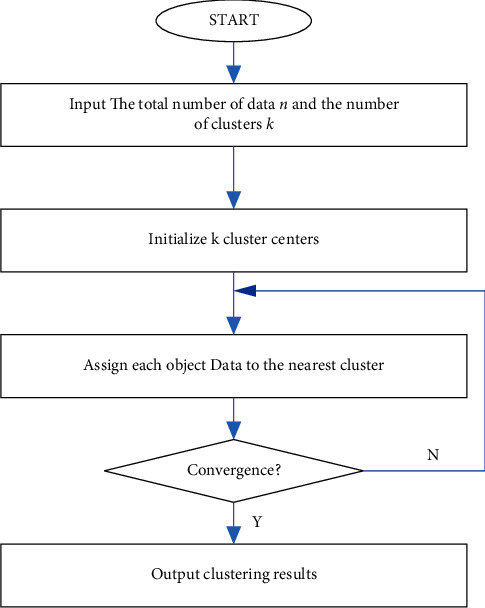
Flow diagram for K-mean cluster.

The flow diagram for K-mean cluster is shown in Figure 4.

(1) $$\begin{array}{c} d\left(x,C_{i}\right)=\sqrt{\sum_{j=1}^{m}{\left(x-C_{i}\right)}^{2}}, \end{array}$$

where *x*is sample data; *C*_*i*_ is the *i* − th cluster center; *m* is the dimension of the data sample; *x*_*j*_ and *C*_ij_ is the *j*-th attribute value of *x* and *C*_*i*_.

### 3.2. Fuzzy C-Means

**Figure 5:**
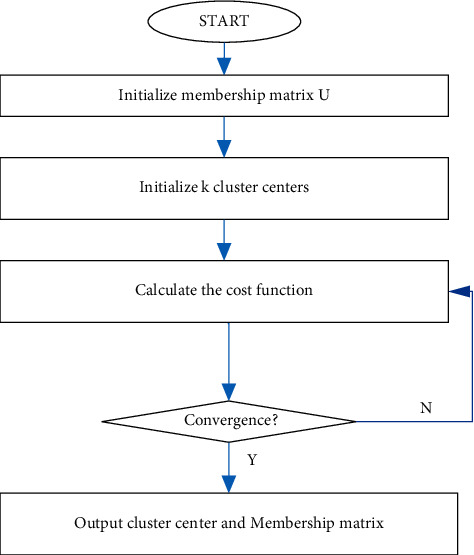
Flow diagram for Fuzzy C-mean cluster.

One of the most popular unsupervised clustering algorithms is called the K-means algorithm. This is due to the fact that it is both efficient and simple to use. It employs an iterative methodology and serves a sizable user population. On the other hand, the K-means algorithm is a challenging approach to clustering that calls for the number of cluster categories and classification groups to be determined in advance. This method has several limitations that must be taken into consideration in the event that there is uncertainty regarding the classification categories. The Fuzzy C-means (FCM) approach is a type of unsupervised data clustering that utilizes membership in order to determine which category a data sample is a part of in order to classify the sample. In contrast to the k-means algorithm, the fuzzy clustering method, also known as FCM, is not as much of a fuzzy clustering method as it is a hard one. When using fuzzy clustering, it is not necessary for each data sample to be distinctly arranged within a single category. On the other hand, as shown in Figure 5, the category can be classified in an infinite variety of different ways. When it comes to dealing with uncertain or hazy categories, the fuzzy clustering method, also known as the FCM, is one of the most prominent lines of clustering development and offers a number of benefits. The following equations can be used to express clustering based on the FCM:

(2) $$\begin{array}{c} \min\;J\left(u,v\right)=\overset{N}{\underset{i=1}{\sum }}\overset{C}{\underset{j=1}{\sum }}u_{ij}^{m}{\left\|x_{i}-x_{j}\right\|}^{2},\\ subjected.t\overset{C}{\underset{j=1}{o.\sum }}u_{ij}=1,u_{ij}\geq 0, \end{array}$$

where *m* is the membership factor, usually taken as 2; ‖*x*_*i*_ − *v*_*j*_‖^2^ represents the Euclidean distance from the current data point to the center point; *x*_*i*_ is the current pixel point, *v*_*j*_ is the cluster center point, *N* is the total number of data points, *C* is the contour curve, and (2) indicates that the sum of membership degrees of all categories is 1.

The process of texture image segmentation can be regarded as the type of sample data belongs to which kinds of textures. For texture images, the pixels have uncertain characteristics. Therefore, it is not appropriate to use the hard clustering method to divide the categories of pixels. FCM can better use the image information and apply the fuzzy relationship to the texture image, which can have a better and more accurate cluster & segmentation effect.

### 3.3. Gaussian Mixture Model

The general expression of Gaussian mixture model can be rewritten as:

(3) $$\begin{array}{c} P\left(x\right)=\sum_{k=1}^{K}\alpha_{k}\varphi \left(x\left|\theta_{k}\right.\right), \end{array}$$

where K represents the number of Gaussian distributions that make up the mixed distribution, i.e., Kclusters; *α*_*k*_ represents the mixing coefficient of the *k* − th cluster, *α*_*k*_ > 0 and ∑_*k*=1_^*K*^*α*_*k*_=1; *φ*(*x*|*θ*_*k*_) represents the Gaussian density, where *θ*_*k*_=(*u*_*k*_, *σ*_*k*_^2^) is denoted as follows:

(4) $$\begin{array}{c} \varphi \left(x\left|\theta_{k}\right.\right)={{\left(2\pi \right)}^{-d/2}\left(\sigma_{k}^{2}\right)}^{-1/2}\;\exp\;\left({\left(x-u_{k}\right)}^{T}/2\sum \left(x-u_{k}\right)\right), \end{array}$$

*u*_*k*_, *σ*_*k*_^2^ represent the mean and covariance matrix of the *k* − th cluster, respectively; *d* represents the data dimension.

Gaussian mixture model adopts EM algorithm to estimate parameters. EM algorithm consists of E-step and M-step. In other words, it maximizes the log likelihood function of incomplete data to estimate parameters of Gaussian mixture model.

The EM algorithm gradually improves the parameters of the model by utilizing the EM steps in a randomized order. This results in a steady increase in the likelihood probability of the parameters as well as the training samples, and the program finally ends at the maximum point.

### 3.4. Mean-Shift

Mean-shift is an approach for density clustering that can be utilized as a way for segmenting texture images. Mean-shift is referred to as “shifting the mean.” The density gradient is utilized in mean-shift in order to estimate the parameters of the samples, and the kernel function is utilized in order to weight the samples. The nonparametric kernel density estimation approach that Silverman suggested offers a methodical demonstration. Mean-shift makes use of the common kernel function principle in order to propose a kernel-based density estimation algorithm. This algorithm assigns weights to samples within each bandwidth in such a way that the contribution of the offset to the mean-shift vector varies depending on the distance between the sample and the offset point. In other words, the kernel-based density estimation algorithm uses the common kernel function principle.

Given *n*sample points *x*_*i*_, *i*=1,2,…, *n* in the *d* dimensional space *R*^*d*^, the mean-shift vector at the points can be written as follows:

(5) $$\begin{array}{c} M_{h}\left(x\right)=\frac{1}{k}\underset{x_{i}\in S_{h}}{\sum }\left(x_{i}-x\right), \end{array}$$

The Mean-shift algorithm is extended to the following form:

(6) $$\begin{array}{c} M_{h}\left(x\right)=\frac{\sum_{i=1}^{n}G\left(\left(x_{i}-x\right)/h\right)w\left(x_{i}\right)\left(x_{i}-x\right)}{\sum_{i=1}^{n}G\left(\left(x_{i}-x\right)/h\right)w\left(x_{i}\right)}, \end{array}$$

where *w*(*x*) ≥ 0 is the weight of sample point *x*; *G*(*x*_*i*_) is a unit kernel function, *G*(*x*)=*g*(‖*x*^2^‖); *h* is the bandwidth of the kernel function.

### 3.5. Fuzzy Subspace Clustering

In order to cluster high-dimensional data sets, a new approach called fuzzy subspace clustering (FSC) has been devised. This algorithm was inspired by fuzzy clustering and LAC. The FSC algorithm locates the subspace clusters in which every dimension of the initial data is linked to each cluster in terms of the probability or weight. In a dimension, the weight that is to be attributed to the dimension is proportional to the cluster density. The higher the cluster density, the bigger the weight. In other words, each cluster has an association with all dimensions of the data that was initially collected.

One of the subcategories of clustering methods is referred to as fuzzy subspace clustering. In addition to having strong denoising capabilities, it is capable of transforming high-dimensional data into useful subspaces for clustering. The following is an expression that can be used to describe the objective function of fuzzy subspace clustering:

(7) $$\begin{array}{c} \left.J_{FSC}=\sum_{k=1}^{K}\sum_{i=1}^{d}w_{ki}^{T}\left(u_{kj}\right.{\left(x_{ji}-v_{ki}\right)}^{2}+\varepsilon_{0}\right),\\ subjected.to.u_{kj}\in \left\{0,1\right\},\sum_{k=1}^{K}u_{kj}=1;0<\sum_{j=1}^{n}u_{kj}<N,\\ 0\leq w_{ki}\leq 1;\sum_{i=1}^{d}w_{ki}=1, \end{array}$$

where *K*, *N,* and *d* represent the number of clusters, the number of samples and the characteristic dimension of samples, respectively; *ε*_0_ is a small regularization constant. In order to reflect the characteristics of subspace clustering, the weight vector *w*_*k*_, *k*=1,2,…, *K* for each cluster is designed, where *ω*_ki_ ∈ *ω*_*k*_ indicates the contribution of the *i*th feature to the *k*-th cluster.

Given training data set *D*_*tr*_=(*x*_*i*_, *y*_*i*_), *x*_*i*_ ∈ *R*^*d*^, *y*_*i*_ ∈ *R*, *i*=1,2,…, *N*, *X*={*x*_1_, *x*_2_,…, *x*_*N*_} is divided into *k* classes by FSC algorithm, which is corresponding to K fuzzy rules. Each feature selected by each fuzzy rule corresponds to a fuzzy subset *A*_*i*_^*k*^. If the Gaussian function is used as the membership function, the corresponding Gaussian function parameters for *A*_*i*_^*k*^ can be estimated as follows:

(8) $$\begin{array}{c} c_{i}^{k}=\sum_{j=1}^{N}u_{kj}x_{ji}^{k}/\sum_{j=1}^{N}u_{kj},\\ \delta_{i}^{k}=h\sum_{j=1}^{N}u_{kj}\left(x_{ji}^{k}-c_{i}^{k}\right)/\sum_{j=1}^{N}u_{kj}, \end{array}$$

where *x*_ji_^*k*^ denotes the *i*th feature selected by the *j*-th sample *x*_*j*_=(*x*_*j*1_, *x*_*j*2_,…,*x*_*j* *d*_)^*T*^ from the *k*-th fuzzy rule; *u*_kj_ represents whether the *j*-th sample belongs to the *K*-th cluster.

### 3.6. Maximum Entropy Clustering Algorithm

The Maximum Entropy Clustering Algorithm (also known as MEC) is one of the clustering algorithms that is considered to be among the most representative. The mathematical representation of it is straightforward, and the physical significance of what it means is unmistakable. It is an algorithm for clustering that is frequently employed by academics. The MEC clustering algorithm has better denoise than the classical fuzzy C-means clustering, which makes it possible to obtain better clustering and brings the segmentation results closer to the ground-truth results. This is especially useful in the segmentation of texture images that contain noise. The expression of the function that the MEC algorithm uses is as follows:

(9) $$\begin{array}{c} \min_{U,V}\left(\overset{C}{\underset{i=1}{\sum }}\overset{N}{\underset{j=1}{\sum }}u_{ij}{\left\|x_{j}-V_{i}\right\|}^{2}+\lambda \overset{C}{\underset{i=1}{\sum }}\overset{N}{\underset{j=1}{\sum }}u_{ij}\ln u_{ij}\right),\\ subjected.to.0\leq u_{ij}\leq 1\overset{C}{\underset{i=1}{\sum }}u_{ij}=1,1\leq i\leq C,1\leq j\leq N. \end{array}$$

## 4. Comparative Performance Analysis

Traditional methods have been refined over the course of many decades, which has resulted in their high level of maturity and widespread application in engineering. The primary objective of this study is to evaluate and assess a wide range of existing typical texture classification algorithms, evaluate and assess their performance with regard to image size, clustering number, running time, and accuracy, and determine the performance boundaries of various algorithms in order to determine the focus for subsequent improved algorithms.

### 4.1. Data Sources for Texture Classification

**Figure 6:**
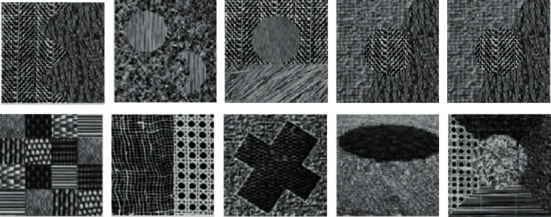
The sample of ten representative Brodatz texture images.

In this study, the Brodatz texture images found in the public database serve as the basis for feature extraction and classification exercises involving a variety of texture clustering techniques. 112 distinct grayscale texture images are included in the Brodatz texture dataset. Each image has a resolution of 640 pixels by 640 pixels and 8 bits.  Figure 6 depicts the 10 representative texture images that were chosen for the study, and they are available for download at http://www.ux.uis.no/tranden/brodatz.html. This selection was made to make the analysis more manageable. The Brodatz texture image that we have chosen is a composite image that is made up of seven individual basic texture images, and the size of the composite image has been resized to match the size of the individual basic texture photographs. In order to create an accurate representation of the environment in which the real dataset was collected, Gaussian noise with varying standard deviations was applied to each texture image.

For the purpose of the experiment, each texture image with a resolution of 640 by 640 pixels needs to be segmented into sixteen nonoverlapping subimages of either 100 by 100 or 124 by 124 pixels. To put that another way, the experiment requires a library of texture images with 640 different subimages, but we only chose 10 representative photos to analyze. In accordance with the experimental steps, the feature of each sub-image is extracted. In the first step, a Gabor filtering preprocessing operation is carried out on the grayscale image, and this operation extracts the features of the image's texture.

### 4.2. Experimental Setup

#### 4.2.1. Comparison Algorithm

After decades of growth, the traditional technique has reached a high level of maturity and has seen widespread use in the field of engineering. In the area of texture feature extraction, researchers have made significant strides, leading to the development of new algorithms such as K-means clustering algorithm, Fuzzy C-Means algorithm, Gaussian mixture model, Mean-Shift, Fuzzy Subspace clustering, and Maximum Entropy Clustering Algorithm. All of these algorithms were proposed by the researchers. The theory behind several algorithms as well as the technique of putting them into practice was presented in Section 2 of this study. It is important to note that all of the comparison algorithms used for this research employ open source MATLAB code, and the default values for their parameters. This makes quantitative analysis of following trials more convenient. The operational system is a 32 bit version of Windows 10, and the programming environment is MATLAB 7.10.0.499. The experimental hardware consists of an Intel Core i5-7240 CPU, which has a basic frequency of 3.40 GHz and 4 GB of memory (R2017a).

#### 4.2.2. Evaluation Criteria

In order to quantitatively evaluate the performance of different comparison texture clustering algorithms, the experiment adopts running time, Rand index (RI) and Normal Mutual Information (NMI) for quantitative analysis. The calculation equation of RI and NMI is shown in the following equations:.

(10) $$\begin{array}{c} \text{NMI}=\frac{\sum_{i=1}^{c}\sum_{j=1}^{c}\left(N_{ij}\log\;N\cdot N_{ij}\right)/N_{i}\cdot N_{j}}{\sqrt{\sum_{i=1}^{c}N_{i}\log N_{i}/N}\sqrt{\sum_{j=1}^{c}N_{j}\log N_{j}/N}}, \end{array}$$

(11) $$\begin{array}{c} \text{RI}=\frac{f_{00}+f_{11}}{N\left(N-1\right)/2}. \end{array}$$

### 4.3. Performance Analysis for Different Texture Clustering Algorithms

In our experiment, we begin by applying Gabor preprocessing to the texture image in order to reduce the amount of interference from the surrounding noise. Based on the preprocessed data, we next employ a variety of approaches to extract features and cluster texture. With the Gabor filter, you can accurately depict and identify various textures. Using a Gaussian kernel function in the spatial domain, a two-dimensional Gabor filter is equivalent. Because of the multiplicative convolution feature, the Fourier transform of the Gabor filter impulse response is a convolution of the harmonic function Fourier transform and the Gaussian function Fourier transform. In order to construct the filter, two parts must be used: a real one and an imaginary one that are orthogonal to one another. As a result, there are three stages to the texture clustering process. The first step in the clustering process is to perform picture preprocessing using the Gabor method to reduce the amount of noise in the image. Extracting texture feature vectors from each pixel in an image, and then using these vectors to generate picture features, is the second phase. The final clustering results are obtained in the third stage, which involves applying a variety of clustering approaches. The texture feature vectors of each pixel in the image are processed using these techniques. It is possible to produce a clustered image by separating pixels into multiple clusters and then using the texture clustering to display the resulting image with different gray values for each of the different clusters. As part of our evaluation of the various texture clustering algorithms, we will undertake both qualitative and quantitative data analysis.

**Figure 7:**
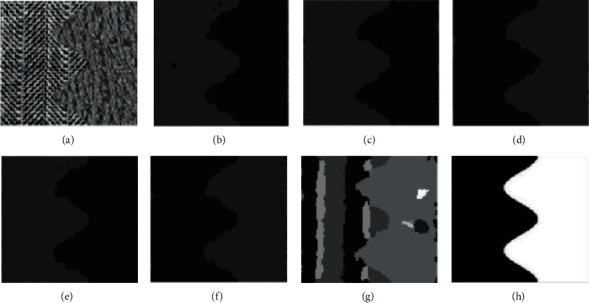
Clustering results of different algorithms in texture image t21. (a) Raw image. (b) FCM. (c) FSC. (d) GMM. (e) MEC. (f) K-means. (g) Mean-shift. (h) Ground-truth.

**Figure 8:**
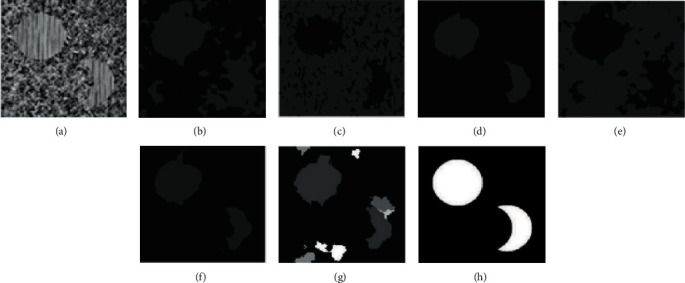
Clustering results of different algorithms in texture image t22. (a) Raw image. (b) FCM. (c) FSC. (d) GMM. (e) MEC. (f) K-means. (g) Mean-shift. (h) Ground-truth.

**Figure 9:**
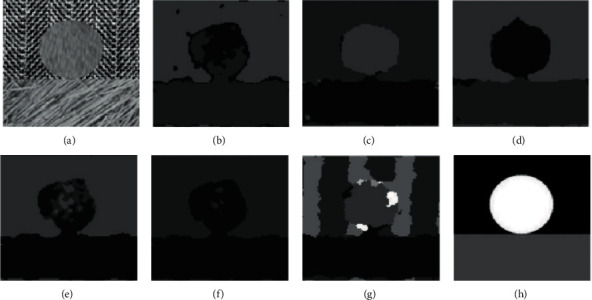
Clustering results of different algorithms in texture image t31. (a) Raw image. (b) FCM. (c) FSC. (d) GMM. (e) MEC. (f) K-means. (g) Mean-shift. (h) Ground-truth.

**Figure 10:**
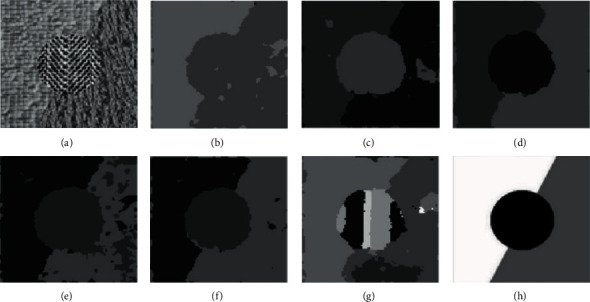
Clustering results of different algorithms. (a) Raw image. (b) FCM; (c) FSC. (d) GMM. (e) MEC. (f) K-means. (g) Mean-shift. (h) Ground-truth.

**Figure 11:**
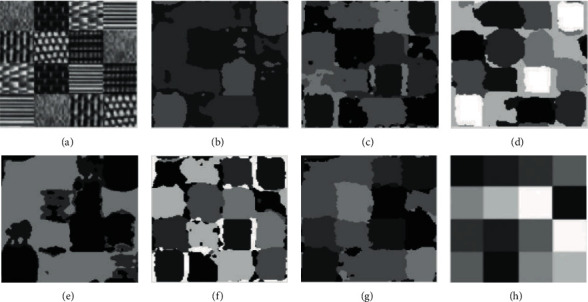
Clustering results of different algorithms. (a) Raw image. (b) FCM. (c) FSC. (d) GMM. (e) MEC. (f) K-means. (g) Mean-shift. (h) Ground-truth.

In comparative experiment, we selected 10 representative images for analysis. However, due to space limitations, this section only selects the clustering results of 6 texture images for qualitatively discussing the results. Figures 7–11 are the clustering results of different algorithms for different texture images.

Figure 7 is the clustering result of the texture image t21. It can be clearly seen from the raw image that the texture image should be divided into two categories, but except that the results of mean-shift are messy, FCM, FSC, GMM, MEC, and K-means can get more accurate results. The boundary of FSC and GMM is smooth, while other results are rough and misclassified. The essence of the two-dimensional K-means model is that it draws a circle with the center of each cluster. In other words, the center of the circle is the maximum Euclidean distance from the center of a cluster to the center of another cluster as the radius. That means it truncates the training set by a circle. Moreover, K-means requires that the shape of these clusters must be circular. Therefore, the cluster fitted by K-means model is very different from the actual data distribution (maybe ellipse), and multiple circular clusters are often mixed and overlapped with each other. In general, K-means has two shortcomings, which makes its fitting effect on many data sets (especially low dimensional data sets) unsatisfactory: the shape of texture is not flexible enough, the fitting results are quite different from the actual results, and the accuracy is limited.

Figure 8 is a clustering result of the texture image t22. It can be seen that the raw image has three textures, and the difference is small, GMM and K-means can get more accurate results, while FCM, FSC, MEC, and mean-shift have poor clustering results. There are a large number of misclassification phenomena in the background region of FCM. From the results, it can be clearly seen that FCM algorithm divides the upper and lower triangular regions very disorderly. MEC and mean-shift classify the two regions into one class in the texture image.

Figure 9 is a clustering result of the texture image t31. It is obvious in the result that although FCM algorithm has segmented three categories in texture clustering, there is a problem in the segmented region. The upper rectangle and the lower rectangle are not accurately segmented. However, compared with the segmentation results of FSC and MEC algorithm, the segmentation accuracy of FCM algorithm is improved. From the results in Figure 9(f), it can be seen that the k-means algorithm directly classifies the regions with very low density as the background, while the FCM algorithm clearly divides the regions with low density. Therefore, FCM algorithm can deal with uneven density region well.

Figure 10 is a clustering result of the texture image. From the clustering results, it can be seen that FSC and MEC algorithms classify the edges of two adjacent classes into two classes, K-means algorithm segments the edges and background together, and FCM algorithm classifies the edges and squares into one class. So FCM algorithm can also deal with the uneven edge region.

As can be seen from the clustering results in Figure 11, although the FSC and MEC algorithms segment the texture of the middle region, they do not accurately segment the texture of the edge region. Figure 11 is a square region with multiclass textures. From the ground-truth clustering results, FCM, FSC, GMM, and K-means can be used in some grid regions, but the results are not ideal. In particular, the category of boundary area is not accurate, resulting in unclear boundary. Mean-shift divides several different textures into the same category.

According to the above clustering results, compared with the image segmentation results of K-means algorithm, FCM, FSC, and mean-shift algorithm, GMM algorithm can reduce the number of wrong segmentation regions and obtain good segmentation results. Gaussian mixture model (GMM) can be regarded as an optimization of K-means model. It is not only a common technical means in industry, but also a generative model. Gaussian mixture model tries to find the mixed representation of multi-dimensional Gaussian probability distribution, so as to fit arbitrary shape data distribution.

In order to quantitatively evaluate the performance of different comparison texture clustering algorithms, the experiment adopts running time, Rand index (RI) and normal mutual information (NMI) for quantitative analysis. Tables 1–3 show the comparative analysis for running time, rand index, and normalized mutual information.  Table 1 shows the effect of image size on the running-time results. Two different sizes of texture images are selected in the experiment. For texture image with a size of 124 × 124, the running time of FCM is 22.85, while the image processing time of 100 × 100 is 18.69. In general, the running time of 124 × 124 pixels image is longer than that of 100*∗*100 for any algorithm. From this, we can conclude that the size of the image determines the length of the running time. The larger the size of image, the slower the running time.

In this experiment, a variety of experiments are carried out on the number of clusters. Five synthetic texture images with two clusters, two images with three clusters, one image with four clusters, one image with five clusters, and one image with seven clusters are selected. For example, there are seven clusters in D1. As for the RAND index, when the number of clusters is more, the value of the RAND index is inversely proportional. In other words, the more the number of clusters, the lower the value of the RAND index. In normalized mutual information, we can also see that when the number of clusters is greater, the value of normalized mutual information is inversely proportional. The more the number of clusters, the lower the value of NMI as shown in Tables 1-2.

In texture image D1 with the size of 124×124, it can be seen that GMM algorithm and K-means algorithm have better effects. For the three evaluation indicators, GMM segmentation has the best effect. It can be seen from Table 1 that MEC algorithm has the fastest running time, but the GMM algorithm has the best clustering results for Rand index and normalized mutual information, but MEC is not the worst. Therefore, the correlation between running time and clustering performance index is not strong. By comparing result of five images with two clusters and result of two images with three clusters, it is concluded that when the image texture is relatively independent, it will be easier to segment. When a texture in image contains another texture, it will cause large errors and cause misclassification.

Through the data analysis in Tables 2 and 3, each algorithm can find that there is a proportional relationship between Rand index and normalized mutual information, which is the embodiment of the clustering performance. When one value is larger, the other is larger, and the value of rand index is larger than that of normalized mutual information.

## 5. Conclusion and the Future Work

Traditional texture cluster algorithms have seen widespread application in engineering, but despite this, there are ongoing challenges associated with their high complexity and limited universal applicability. This paper focuses primarily on the analysis of the performance of various texture clustering algorithms. It also compares the performance of traditional texture classification algorithms in terms of image size, clustering number, running time, and accuracy. Finally, it determines the performance boundaries of various algorithms in order to locate the focus for subsequent improved algorithms. For the purposes of the experiment, a number of traditional clustering algorithms have been chosen to serve as benchmarks for evaluating overall performance. Both qualitative and quantitative findings point to the fact that the performances of various algorithms are quite distinct from one another. Better performance can only be achieved by selecting the appropriate algorithm in accordance with the characteristics of the image's texture. In the future, we are going to conduct an analysis of the texture segmentation algorithm that is based on deep learning. In addition, we are going to conduct an in-depth comparison of the conventional algorithm and the intelligent algorithm in order to determine the conditions under which they can adapt and the boundaries of their performance.

## Figures and Tables

**Table 1:** Comparative analysis for running time.

Images	Size	Cluster	FCM	FSC	GMM	MEC	Kmeans	Mean-shift
T21	100 × 100	2	34.51	6.32	7.50	3.26	3.55	15.93
T22	100 × 100	2	9.11	5.59	8.10	3.42	3.45	28.42
T31	100 × 100	3	21.99	5.64	16.84	4.39	5.94	9.77
T32	100 × 100	3	18.69	7.74	8.29	4.07	4.58	15.45
T41	100 × 100	4	18.69	10.40	58.70	6.87	6.99	11.74
D1	124 × 124	7	22.85	23.34	192.58	17.77	17.16	24.93
Z2	100 × 100	5	34.13	14.24	62.53	13.12	13.30	28.61
Z3	100 × 100	2	23.65	4.34	10.39	3.74	3.44	13.62
Z4	100 × 100	2	13.87	4.21	9.93	3.93	4.06	16.45
Z5	100 × 100	2	7.62	3.25	20.93	2.73	3.42	4.80

**Table 2:** Comparative analysis for Rand Index (RI).

Images	Size	Cluster	FCM	FSC	GMM	MEC	Kmeans	Mean-shift
T21	100 × 100	2	0.92	0.95	0.97	0.96	0.96	0.76
T22	100 × 100	2	0.74	0.73	0.91	0.74	0.91	0.84
T31	100 × 100	3	0.84	0.88	0.91	0.81	0.78	0.80
T32	100 × 100	3	0.79	0.91	0.92	0.84	0.93	0.85
T41	100 × 100	4	0.61	0.91	0.92	0.79	0.91	0.88
D1	124 × 124	7	0.66	0.72	0.76	0.62	0.76	0.71
Z2	100 × 100	5	0.71	0.75	0.85	0.66	0.74	0.80
Z3	100 × 100	2	0.72	0.72	0.74	0.72	0.70	0.70
Z4	100 × 100	2	0.76	0.73	0.77	0.80	0.81	0.59
Z5	100 × 100	2	0.90	0.91	0.87	0.91	0.94	0.93

**Table 3:** Comparative analysis for normalized mutual information (NMI).

Images	Size	Cluster	FCM	FSC	GMM	MEC	Kmeans	Mean-shift
T21	100 × 100	2	0.76	0.85	0.88	0.85	0.86	0.52
T22	100 × 100	2	0.39	0.31	0.67	0.40	0.66	0.49
T31	100 × 100	3	0.56	0.70	0.74	0.55	0.53	0.55
T32	100 × 100	3	0.58	0.73	0.78	0.61	0.79	0.61
T41	100 × 100	4	0.34	0.74	0.75	0.51	0.71	0.64
D1	124 × 124	7	0.31	0.36	0.50	0.30	0.48	0.42
Z2	100 × 100	5	0.43	0.48	0.51	0.33	0.48	0.44
Z3	100 × 100	2	0.36	0.45	0.49	0.35	0.33	0.37
Z4	100 × 100	2	0.48	0.45	0.51	0.49	0.52	0.34
Z5	100 × 100	2	0.70	0.72	0.68	0.76	0.80	0.77

## Data Availability

The dataset used to support the findings of this study are available from the corresponding author upon request.
